# Vaccine coverage by social strata in state capitals in the Brazilian Midwest region: a household survey of children born in 2017 and 2018

**DOI:** 10.1590/S2237-96222024v33e20231308.especial2.en

**Published:** 2024-11-01

**Authors:** Jaqueline Costa Lima, Érica Marvila Garcia, Sandra Maria do Valle Leone de Oliveira, Wildo Navegantes de Araújo, Emmanuela Maria de Freitas Lopes, Sheila Araújo Teles, Karlla Antonieta Amorim Caetano, Ana Izabel Passarela Teixeira, Bárbara Manuella Cardoso Sodré Alves, Ana Paula França, José Cássio de Moraes, Carla Magda Allan Santos Domingues, ICV 2020 Group, Adriana Ilha da Silva, Alberto Novaes Ramos, Ana Paula França, Andrea de Nazaré Marvão Oliveira, Antonio Fernando Boing, Carla Magda Allan Santos Domingues, Consuelo Silva de Oliveira, Ethel Leonor Noia Maciel, Ione Aquemi Guibu, Isabelle Ribeiro Barbosa Mirabal, Jaqueline Caracas Barbosa, Jaqueline Costa Lima, José Cássio de Moraes, Karin Regina Luhm, Karlla Antonieta Amorim Caetano, Luisa Helena de Oliveira Lima, Maria Bernadete de Cerqueira Antunes, Maria da Gloria Teixeira, Maria Denise de Castro Teixeira, Maria Fernanda de Sousa Oliveira Borges, Rejane Christine de Sousa Queiroz, Ricardo Queiroz Gurgel, Rita Barradas Barata, Roberta Nogueira Calandrini de Azevedo, Sandra Maria do Valle Leone de Oliveira, Sheila Araújo Teles, Silvana Granado Nogueira da Gama, Sotero Serrate Mengue, Taynãna César Simões, Valdir Nascimento, Wildo Navegantes de Araújo

**Affiliations:** 1 Universidade Federal de Mato Grosso, Faculdade de Enfermagem, Cuiabá, MT, Brazil; 2 Secretaria de Saúde de Marataízes, Vigilância em Saúde, Espírito Santo, ES, Brazil; 3 Fundação Oswaldo Cruz, Campo Grande, MS, Brazil; 4 Universidade de Brasília, Faculdade de Ceilândia, Brasília, DF, Brazil; 5 Secretaria Municipal de Saúde, Programa de Pós-graduação em Doenças Infecciosas e Parasitárias, Campo Grande, MS, Brazil; 6 Universidade Federal de Goiás, Goiânia, GO, Brazil; 7 Universidade Federal de Mato Grosso do Sul, Paranaíba, MS, Brazil; 8 Laboratório de Estudos Farmacêuticos, Universidade de Brasília, Brasília, DF, Brazil; 9 Faculdade de Ciências Médicas da Santa Casa de São Paulo, Departamento de Saúde Coletiva, São Paulo, SP, Brazil; 10 Organização Pan-Americana da Saúde, Brasília, DF, Brazil; Universidade Federal do Espírito Santo, Vitória, ES, Brazil; Universidade Federal do Ceará, Departamento de Saúde Comunitária, Fortaleza, CE, Brazil; Faculdade Ciências Médicas Santa Casa de São Paulo, São Paulo, SP, Brazil; Secretaria de Estado da Saúde do Amapá, Macapá, AP, Brazil; Universidade Federal de Santa Catarina, SC, Brazil; Organização Pan-Americana da Saúde, Brasília, DF, Brazil; Instituto Evandro Chagas, Belém, PA, Brazil; Faculdade de Ciências Médicas Santa Casa de São Paulo, Departamento de Saúde Coletiva, São Paulo, SP, Brazil; Universidade Federal de Mato Grosso, Cuiabá, MT, Brazil; Universidade Federal do Paraná, Curitiba, PR, Brazil; Universidade Federal de Goiás, Goiânia, GO, Brazil; Universidade Federal do Piauí, Teresina, PI, Brazil; Universidade de Pernambuco, Faculdade de Ciências Médicas, Pernambuco, PE, Brazil; Instituto de Saúde Coletiva, Universidade Federal da Bahia, Salvador, BA, Brazil; Secretaria de Estado da Saúde de Alagoas, Maceió, AL, Brazil; Universidade Federal do Acre, Rio Branco, AC, Brazil; Universidade Federal do Maranhão, Departamento de Saúde Pública, São Luís, MA, Brazil; Universidade Federal de Sergipe, Aracaju, SE, Brazil; Secretaria Municipal de Saúde, Boa Vista, RR, Brazil; Fundação Oswaldo Cruz, Mato Grosso do Sul, Campo Grande, MS, Brazil; Fundação Oswaldo Cruz, Escola Nacional de Saúde Pública Sergio Arouca, Rio de Janeiro, RJ, Brazil; Universidade Federal do Rio Grande do Sul, Porto Alegre, RS, Brazil; Fundação Oswaldo Cruz, Instituto de Pesquisa René Rachou, Belo Horizonte, MG, Brazil; Secretaria de Desenvolvimento Ambiental de Rondônia, Porto Velho, RO, Brazil; Universidade de Brasília, Brasília, DF, Brazil

**Keywords:** Programas de Inmunización, Cobertura vacunal, Factores socioeconómicos, Desigualdades Sociales en Salud, Encuestas de Población, Immunization Programs, Vaccination Coverage, Socioeconomic Factors, Socioeconomic Disparities in Health, Population Surveys

## Abstract

**Objective:**

To analyze full vaccination coverage in live births in 2017 and 2018 in the capitals of the Midwest region of Brazil, according to social strata.

**Methods:**

Population-based household survey with cluster sampling. Full coverage in children at 12 and 24 months of age and sociodemographic factors were analyzed.

**Results:**

5,715 children were analyzed. Full coverage at 12 months of age was 67.9% (95%CI 65.4;70.4), while at 24 months it was 48.2% (95%CI 45.3;51.1). Pneumococcal vaccine had the highest vaccination coverage (91.3%), while the second dose of rotavirus vaccine had the lowest (74.2%). In Campo Grande, no vaccine reached coverage above 90%, with BCG (82.9%) and hepatitis B (82.1%) standing out. Campo Grande and Brasília had the worst vaccination coverage in the high social stratum (24 months of age).

**Conclusion:**

Vaccination coverage in the Midwest was below 80%, falling short of the recommended target and associated with socioeconomic factors.

## Study contributions

**Main results:**

Vaccination coverage at 24 months old among the Midwest state capitals ranged between 39.9% in Campo Grande and 54.5% in Brasília. All vaccination coverage was below the target recommended by the National Immunization Program.

**Implications for services:**

The results found point to the urgent need for planning actions aimed at improving vaccination coverage with targeted approaches, considering social strata and vaccines with lower vaccination coverage.

**Perspectives:**

Future research that investigates vaccination coverage in the Midwest region may assist in understanding the low vaccination coverage found for most recommended vaccines, especially in the first 24 months of life.

## RESUMEN

**Objetivo:**

Evaluar la cobertura vacunal completa en nacidos vivos en 2017 y 2018, en las capitales de la región Centro-Oeste de Brasil, según estrato social.

**Métodos:**

Encuesta poblacional de hogares con muestreo por conglomerados. Se analizó la cobertura vacunal completo de niños de 12 y 24 meses de edad e indicadores sociodemográficos.

**Resultados:**

Se analizaron 5.715 niños. La cobertura vacunal completa a los 12 meses de edad fue de 67,9% (IC_95_% 65,4;70,4) y a los 24 meses de edad fue de 48,2% (IC_95_% 45,3;51,1). La cobertura vacunal más alta fue de la vacuna antineumocócica (91,3%) y la más baja fue la segunda dosis de la vacuna contra rotavirus (74,2%). En Campo Grande, ninguna vacuna logró coberturas superiores al 90%, destacándose la vacuna BCG (82,9%) y la hepatitis B (82,1%). Campo Grande y Brasilia tuvieron las peores coberturas vacunales en el estrato social alto (24 meses de edad).

**Conclusión:**

La cobertura vacunal en la región Centro-Oeste fue inferior al 80%, por debajo de la meta recomendada y asociada a factores socioeconómicos.

## INTRODUCTION

Reduction in vaccination coverage (VC) has been seen worldwide in the last decade.^1^ Despite the existence of a Global Vaccine Action Plan, proposed by the World Health Organization (WHO) in 2022, around 14 million children living in low- and middle-income countries, such as Angola, Brazil, Democratic Republic of Congo, Ethiopia, India, Indonesia, Mozambique, Nigeria, Pakistan and the Philippines, did not complete their vaccination schedule.^2^

In Brazil, the drop in vaccination coverage began in 2012, and worsened during the COVID-19 pandemic.^3^ Between 2019 and 2021, the 90% desired vaccination coverage for DPT, measles and pneumococcal vaccination was not achieved,^3^ this being an important global monitoring indicator defined by the 2030 Immunization Agenda.^4^ The cause of such a reduction in vaccination coverage is multifaceted and requires an understanding of public health interventions in Brazil, operability of actions, as well as the regional, national and international geographic and political context.^5^

The history of vaccination implementation in Brazil dates back to the beginning of the 19^th^ century, when the gradual and free introduction of immunobiological products took place. The creation of the National Immunization Program (PNI), in 1973, was a milestone for public health in Brazil and changed the epidemiological scenario of transmissible infections throughout the country. After the introduction of the program, systematized actions to eradicate vaccine-preventable diseases began, with the expansion of the supply of vaccines to the entire Brazilian population.^5,6^

Despite significant reductions in social disparities and improvements in health indicators over the last few decades in Brazil, administrative data show intra-regional and inter-regional differences in vaccination coverage in different regions of the country. On the other hand, validating these results through household surveys is desirable, since previous studies have shown large discrepancies between administrative data and survey data.^7,8^

As such, more than ten years after the last vaccination coverage household survey carried out in the Brazilian state capital cities,^9^ there is a need to understand the current panorama of vaccination coverage in the Midwest region of the country which, although it is a strategic region, is lacking in scientific production.^10^ We believe that validating these data will allow us to identify possible opportunities for improvements in vaccination coverage indicators, generate hypotheses for new research and expand knowledge about the factors that may be related to vaccination. The objective of this study was therefore to analyze vaccination coverage among children born in 2017 and 2018, in the state capitals of the Midwest region of Brazil, according to social strata. 

## METHODS

### Study design 

This is a population-based survey, using data from the Vaccination Coverage Survey (Inquérito de Cobertura Vacinal - ICV), on valid dose vaccination coverage in the Midwest region of Brazil. The ICV survey was conducted in Brazil’s 26 states and Federal District between September 2020 and Mach 2022.^11^

### Background 

The Midwest region is Brazil’s second largest region in terms of territorial extension and has an estimated population of 16.3 million inhabitants and a population density that varies from 97.22 inhabitants/km^2^ in Campo Grande to 1,776.8 inhabitants/km^2^ in Goiânia (Box 1).^12^ Its economy is based on agriculture, livestock and mineral extraction, with significant growth in several sectors.^13^

**Box 1:** Sociodemographic characteristics of the families, mothers and children taking part in the four state capital cities of the Midwest region, Brazil, 2020-2022

**Sociodemographic characteristics**	**Cuiabá**	**Campo Grande**	**Goiânia**	**Federal District**
Population^a^	650,912	897,938	1,437,237	2,817,068
Population ≤ 4 years old^a^	43,647	59,766	83,676	166,848
HDI^a^	0.785 (very high)	0.784 (high)	0.799 (very high)	0.824 (very high)
GINI index^b^	0.5293	0.5070	0.4751	0.6370
Per capita GDP^a^	42,918.31	33,243.63	33,826.84	87,016.16
Population density^a^	150.41	111.09	1,970.72	489.01
Infant mortality rate^a^	12.92	10.29	9.26	9.76
Social Vulnerability Index	0.261	0.27	0.291	0.294

a) 2022 census; b) 2010 census.

### Participants

The study population was comprised of children born in 2017 and 2018, who lived in the urban area of the state capitals Campo Grande, Cuiabá and Goiânia, as well as in the urban area of the Federal District. 

### Variables

The study variables were complete and incomplete valid vaccination coverage at 12 and 24 months old, and socioeconomic and demographic characteristics, such as: social stratum (A – high, B – medium, C – low, D – very low) – defined according to head of family income and schooling data;^12^ family consumption level (high, medium, low, very low and did not answer) – defined according to the following cutoff points: high (42 points and more), medium (27-41 points), low (16-26 points) and very low (< 16 points);^14^ household crowding (more than three dwellers sharing the same room used as a bedroom); monthly family income (up to BRL 1,000, BRL 1001 - BRL 3,000, BRL 3,001 - BRL 8,000, more than BRL 8.000, and did not answer); percentage of grandmothers living in the household; maternal characteristics: schooling in years of study (up to 8 years, 9 - 12 years, 13 - 15 years, 16 years or more, unable or refused to answer); age group (< 20 years, 20 - 34 years, 35 years or more, unable or refused to answer); self-reported race/skin color (White, Black, mixed race, Asian, Indigenous, unable or refused to answer); percentage of mothers with paid work, percentage of mothers with a partner; number of children (1 - 3 children, 4 - 7 children and > 7 children); children’s characteristics: sex (female and male); birth order (first, second, third, fourth or more, did not answer); race/skin color (White, Black, mixed race, Asian, Indigenous, unable or refused to answer) and percentage of children attending daycare. 

The following definitions as per Barata et al. (2023) were used in order to analyze vaccination coverage:^11^

- Valid dose: compliance with the schedule in force taking into account the ages recommended by the official PNI schedule and correct intervals between doses.
- Full vaccination coverage for the first 12 months of life (“basic schedule”) consisted of the following vaccines: Bacillus Calmette-Guérin (BCG), hepatitis B, three doses of 5-in-1 vaccine (diphtheria, pertussis, tetanus + *Hemophilus influenzae* type B + hepatitis B) and three doses of inactivated poliovirus vaccine (IPV), two doses each of rotavirus, meningitis C and pneumococcal vaccine, and one dose of yellow fever vaccine.
- Full vaccination coverage at 24 months, included, in addition to the basic schedule vaccines, two doses of MMR (measles, mumps and rubella), one dose each of hepatitis A, chickenpox and bivalent oral poliovirus vaccine (bOPV); and one dose each of DPT booster (diphtheria, pertussis and tetanus), meningitis C, and pneumococcal vaccine.
- When calculating vaccination coverage it was necessary to group together several immunobiological products, as some are only administered in private services, which were also included in the present research.^11^ Vaccines grouped together were described as follows: 5-in-1 vaccination coverage (5-in1, hexavalent and acellular); IPV vaccination coverage (IPV and hexavalent); meningitis C vaccination coverage (meningitis C and meningitis ACWY); MMR vaccination coverage (MMR and tetravalent); chickenpox vaccination coverage (chickenpox and tetravalent); poliomyelitis booster dose (OPV, IPV or hexavalent doses administered after 1 year old – doses not used in the basic IPV schedule); 1^st^ DPT vaccine booster (DPT, 5-in-1, hexavalent or Acel administered after 1 year old and not used in the basic 5-in-1 vaccine schedule).

### Data source 

The data sources we used were the questionnaire prepared to conduct the ICV survey, containing the socioeconomic and demographic variables described above, and photographs of the children’s vaccination cards, containing information about the vaccines administered.^11^

### Sample 

A previously defined complex sample was adopted, depending on the number of live births registered on the Live Birth Information System in 2017 and 2018, the sampling weights for which were calculated for each household interviewed. Initially, basic sampling weights were obtained (inverse of the probabilities of inclusion of the interviewed households), and then these weights were calibrated to known population totals. Two to four surveys were conducted in each state capital city in Midwest region, namely two in Cuiabá, three in Campo Grande and four in Goiânia and Brasília. Refusals, impossibility of conducting the interview after three attempts at different times and on different days and impossibility of locating the expected number of children after an active search throughout the area of ​​the selected clusters were considered to be losses.^11^ The ICV survey operational procedures, sample calculation and other technical information are described in Barata et al., 2023.^11^

### Statistical analysis 

Vaccination coverage (valid doses) was calculated taking the numerator to be those children who received all recommended vaccines in the first year of life (including the yellow fever vaccine), and the denominator to be those children born in 2017 and 2018 included in the study, then multiplying by 100 for the Midwest region state capitals, at 12 and 24 months old, by social stratum. A 95% confidence interval (95%CI) was considered when calculating vaccination coverage. The chi-square test was used to assess the difference between vaccination coverage in the Midwest region state capitals, at 12 and 24 months old, by social stratum. P-values of < 0.05 were considered to be statistically significant. The difference between the vaccination coverage for each vaccine was estimated by subtracting the Midwest region vaccination coverage as a whole from the vaccination coverage of each state capital in the region (referred to as “dif”). We used the Stata (version 17) survey data analysis module to analyze the data. 

### Ethical aspects 

This study was approved by the Human Research Ethics Committee of the Instituto de Saúde Coletiva of the Universidade Federal da Bahia, as per Opinion No. 3.366.818, on June 4^th^ 2019, and as per Certificate of Submission for Ethical Appraisal No. 4306919.5.0000.5030; and by the Human Research Ethics Committee of the Irmandade da Santa Casa de São Paulo, as per Opinion No. 4.380.019, on November 4^th^ 2020, and as per Certificate of Submission for Ethical Appraisal No. 39412020.0.0000.5479. All interviewees signed an informed consent form to be interviewed and signed an authorization for the vaccination cards to be photographed.

## RESULTS

This study included 5,715 children: 31.7% (1,811/5,715) from Goiânia, 31.6% (1,809/5,715) from Brasília, 22.4% (1,281/5,715) from Campo Grande and 14.3% (814/5,715) from Cuiabá. Of the total, 22.7% (1,297) belonged to socioeconomic stratum A, 25.4% (1,451) to stratum B, 25.9% (1,480) to stratum C and 26.0% (1,487) to stratum D. Of the state capitals studied, losses occurred in Cuiabá (9.8%) and in Campo Grande (5.4%). 

The sociodemographic characteristics of the participating families, mothers and children included in this study are presented in Table 1. 

Vaccination coverage for all vaccines in the first 12 and 24 months of life in each of the four participating cities and by social stratum is shown in Table 2. 

**Table 1:** Vaccination coverage at the first 12 and 24 months of life, by social strata, in the four state capitals of the Midwest region of Brazil, 2020-2022

	**Campo Grande** **N (%)**	**Cuiabá** **N (%)**	**Goiânia** **N (%)**	**Brasília** **N (%)**
Number of families included	1,281	814	1,811	1,809
**Social stratum**				
A	271 (20.4)	131 (21.6)	445 (10.9)	450 (8.6)
B	324 (12.9)	226 (13.7)	447 (14.5)	454 (10.6)
C	343 (9.4)	230 (17.4)	452 (20.7)	455 (31.0)
D	343 (57.3)	227 (47.3)	467 (53.9)	450 (49.8)
**Family consumption level**				
High	61 (5.1)	15 (2.2)	37 (1.8)	286 (8.0)
Medium	431 (23.7)	145 (24.1)	509 (16.0)	765 (31.4)
Low	422 (31.2)	342 (33.9)	770 (51.5)	441 (32.3)
Very low	324 (35.0)	295 (37.4)	445 (28.1)	244 (24.6)
Did not answer	43 (5.0)	17 (2.4)	50 (2.6)	73 (3.7)
Household crowding	87 (7.5)	71 (10.2)	59 (6.9)	84 (9.0)
**Monthly family income**				
Up to BRL 1,000	156 (20.1)	196 (23.7)	85 (12.1)	205 (19.6)
BRL 1,001 - BRL 3,000	419 (34.6)	297 (34.6)	631 (41.1)	368 (30.3)
BRL 3,001 - BRL 8,000	404 (25.0)	147 (26.8)	741 (33.1)	370 (21.4)
Above BRL 8,000	172 (10.5)	57 (6.0)	165 (7.1)	689 (22.1)
Did not answer	130 (9.8)	117 (8.9)	189 (6.6)	177 (6.6)
Grandmother living together	327 (29.2)	231 (24.5)	322 (21.1)	449 (31.1)
**Maternal characteristics**				
**Years of schooling**				
Up to 8	85 (10.0)	36 (5.0)	84 (4.5)	60 (4.7)
9 - 12	152 (16.2)	137 (18.5)	215 (13.)	121 (12.3)
13 - 15	435 (35.2)	367 (42.4)	820 (47.7)	483 (37.5)
16 or more	572 (34.0)	257 (31.7)	642 (31.4)	1091 (42.2)
Unable or refused to answer	37 (4.6)	17 (2.4)	50 (2.6)	54 (3.3)
**Age group (years)**				
< 20	35 (4.5)	22 (3.9)	33 (4.9)	20 (3.2)
20 - 34	689 (55.9)	516 (63.1)	1.172 (68.5)	787 (53.0)
35 or over	553 (39.3)	275 (32.8)	583 (25.4)	997 (43.7)
Unable or refused to answer	4 (0.3)	1 (0.2)	23 (1.2)	5 (0.1)
**Mother’s self-reported race/skin color**				
White	690 (46.6)	163 (18.1)	684 (39.6)	838 (36.1)
Black	55 (7.0)	154 (22.8)	117 (8.0)	141 (10.7)
Mixed race	465 (39.5)	473 (55.8)	949 (48.7)	746 (49.7)
Asian	27 (1.6)	8 (0.5)	13 (1.4)	25 (0.8)
Indigenous	6 (0.8)	4 (0.9)	2 (0.4)	6 (0.4)
Unable or refused to answer	38 (4.5)	12 (1.9)	46 (1.9)	53 (2.3)
**Paid work**	779 (56.0)	429 (56.7)	1,087 (52.4)	1,158 (59.2)
**Has a partner**	994 (74.2)	618 (82.5)	1,471 (81.8)	1,457 (75.7)
**Number of children**				
1 - 3	1,159 (87.3)	700 (82.3)	1,650 (89.4)	1,690 (91.3)
4 - 7	120 (12.6)	110 (17.6)	151 (10.4)	112 (8.3)
> 7	1 (0.1)	4 (0.1)	8 (0.2)	2 (0.4)
**Children’s characteristics**				
**Sex**				
Male	651 (53.6)	424 (50.3)	891 (48.4)	909 (50.8)
Female	630 (46.4)	390 (49.7)	920 (51.6)	900 (49.2)
**Birth order**				
First	576 (38.4)	337 (37.9)	841 (44.1)	891 (49.3)
Segundo	441 (35.0)	249 (33.5)	633 (35.3)	621 (30.1)
Third	169 (16.4)	139 (15.6)	212 (13.3)	210 (14.4)
Fourth or more	94 (10.1)	89 (13.0)	124 (6.7)	83 (6.0)
Did not answer	1 (0.02)	0 (0.0)	1 (0.6)	4 (0.2)
**Child’s race/skin color**				
White	834 (59.8)	257 (28.2)	837 (46.9)	1.012 (45.5)
Black	27 (4.1)	91 (11.2)	57 (4.9)	80 (6.4)
Mixed race	396 (34.0)	457 (57.3)	905 (47.0)	687 (47.0)
Asian	16 (1.3)	8 (3.2)	10 (1.1)	16 (0.6)
Indigenous	7 (0.8)	1 (0.04)	1 (0.06)	2 (0.2)
Unable or refused to answer	1 (0.01)	0 (0.0)	1 (0.0)	12 (0.3)
**Attends daycare**	617 (46.0)	372 (45.2)	678 (31.1)	870 (39.8)

Overall valid vaccination coverage for the state capitals of the Midwest region in relation to the vaccine schedule recommended for the first 12 months of life, including doses of yellow fever vaccine, was 67.9% (95%CI 65.4;70.4). When analyzing by participating cities, the highest vaccination coverage was found in Brasília (76.3%) (95%CI 72.5;79.8), while the lowest vaccination coverage was found in Cuiabá (60.4%) (95%CI 54.3;66.3). Contrary to what was observed at 24 months, overall there was no statistical difference in vaccination coverage between social strata. 

Overall valid vaccination coverage for the state capitals of the Midwest region in relation to the vaccine schedule recommended at 24 months, including doses of yellow fever vaccine, was 48.2% (95%CI 45.3;51.1). When analyzing by participating cities, the highest vaccination coverage for the first 24 months of life was found in Brasília (54.5%) (95%CI 49.8;59.1), while the lowest vaccination coverage was found in Campo Grande (39.9%) (95%CI 35.0;45.1) (Table 2).

When considering social strata, a statistical difference in vaccination coverage at 24 months was found for Campo Grande and Brasília. In Campo Grande and Brasília, the lowest vaccination coverage levels were found in stratum A. In Cuiabá and Goiânia, the poorest vaccination coverage was in stratum C (31.8%) (95%CI 23.5;41.5) and stratum B (37.1 %) (95%CI 29.3;45.6), respectively, while there was no statistical significance according to social stratum in Goiânia (Table 2).

Vaccination coverage for each of the vaccines is shown in Table 3. The highest vaccination coverage was found for the first dose of the pneumococcal vaccine (91.3%) and the lowest for the second dose of the rotavirus vaccine (74.2%). We compared vaccination coverage for all the Midwest capitals as a whole in relation to each of the capitals separately. 

**Table 2:** Vaccination coverage updated for vaccine schedule and differences between coverage in the state capitals and coverage in the Midwest region of Brazil, 2020-2022

	**Vaccination coverage at 12 months (%) (95%CI)** ^a^	**p-value** ^b^	**Vaccination coverage at 24 months (%) (95%CI)** ^a^	**p-value** ^b^
**Campo Grande**	**60.7 (54.6;66.5)**		**39.9 (35.0;45.1)**	
**A**	58.4 (44.6;70.9)		25.0 (17.5;34.6)	
**B**	78.1 (65.7;86.8)	0.076	48.3 (38.0;58.7)	0.002
**C**	59.3 (47.9;69.8)		40.4 (31.5;49.2)	
**D**	57.9 (49.6;65.7)		43.4 (36.6;50.4)	
**Cuiabá**	**60.4 (54.3;66.3)**		**46.2 (39.4;53.2)**	
**A**	72.4 (59.4;82.5)		55.9 (42.1;68.9)	
**B**	50.3 (35.7;64.8)	0.055	47.1 (39.1;55.2)	0.050
**C**	53.6 (42.3;64.6		31.8 (23.5;41.5)	
**D**	62.1 (56.2;67.6)		46.8 (37.3;56.5)	
**Goiânia**	**62.1 (57.3;66.6)**		**47.2 (40.8;53.8)**	
**A**	64.7 (44.9;80.5)		43.2 (22.9;66.1)	
**B**	58.2 (51.8;64.4)	0.805	37.1 (29.3;45.6)	0.427
**C**	60.9 (53.6;67.8)		45.8 (41.0;50.8)	
**D**	63.0 (55.7;69.8)		51.3 (40.8;53.8)	
**Brasília**	**76.3 (72.5;79.8)**		**54.5 (49.8;59.1)**	
**A**	75.9 (70.0;80.8)		28.2 (22.7;34.3)	
**B**	73.6 (65.9;80.1)	0.238	29.8 (22.9;37.9)	< 0.001
**C**	80.7 (73.8;86.2)		59.4 (49.5;68.6)	
**D**	74.2 (67.8;79.7)		61.1 (54.3;67.5)	

a) (95%CI): 95% confidence interval; b) Pearson’s chi-square test.

**Table 3:** Vaccination coverage updated for vaccine schedule and differences between coverage in the state capitals and coverage in the Midwest region of Brazil, 2020-2022

Vaccines	Cities								Midwest Region	PNI Target	Dif^a^
	Brasília		Campo Grande		Cuiabá		Goiânia				
	Vaccination coverage (%)	Dif^a^	Vaccination coverage (%)	Dif^a^	Vaccination coverage (%)	Dif^a^	Vaccination coverage (%)	Dif^a^	Vaccination coverage (%)	Vaccination coverage (%)	
BCG	90.9	2.5	82.9	-5.5	90.0	1.6	89.1	0.7	88.4	90	-1.6
Hepatitis B at birth	89.9	2.5	82.1	-5.3	88.9	1.5	87.9	0.5	87.4	95	-7.6
Pentavalent (1^st^ dose)	90.6	-0.1	88.8	-1.9	92.5	1.8	91.4	0.7	90.7	95	-4.3
Pentavalent (2^nd^ dose)	90.1	1.0	86.2	-2.9	90.4	1.3	89.5	0.4	89.1	95	-5.9
Pentavalent (3^rd^ dose)	88.8	2.6	82.5	-3.7	86.1	-0.1	86.3	0.1	86.2	95	-8.8
Inactivated poliovirus vaccine (1^st^ dose)	91.2	0.3	89.7	-1.2	91.8	0.9	91.1	0.2	90.9	95	-4.1
Inactivated poliovirus vaccine (2^nd^ dose)	90.4	0.9	87.6	-1.9	90.5	1.0	89.5	0.0	89.5	95	-5.5
Inactivated poliovirus vaccine 3 (3^rd^ dose)	89.1	2.0	84.3	-2.8	81.1	-6.0	87.0	-0.1	87.1	95	-7.9
Rotavirus (1^st^ dose)	88.5	1.6	85.9	-1.0	87.6	0.7	85.8	-1.1	86.9	95	-8.1
Rotavirus (2^nd^ dose)	80.4	6.2	74.6	0.4	72.2	-2.0	68.7	-5.5	74.2	90	-15.8
Meningococcal C (1^st^ dose)	91.4	0.3	89.6	-1.5	93.2	2.1	91.0	-0.1	91.1	95	-4.4
Meningococcal C (2^nd^ dose)	89.6	1.2	86.6	-1.8	88.0	-0.4	88.7	0.3	88.4	95	-6.6
Meningococcal C (3^rd^ dose)	77.5	0.4	72.8	-4.4	78.9	1.8	79.0	1.9	77.1	95	-17.9
Pneumococcal (1^st^ dose)	91.4	0.1	89.9	-1.4	93.2	1.9	91.2	-0.1	91.3	95	-3.7
Pneumococcal (2^nd^ dose)	90.8	1.0	88.1	-1.7	90.5	0.7	89.7	-0.1	89.8	95	-5.2
Pneumococcal (3^rd^ dose)	66.8	-6.0	69.4	-3.4	81.2	8.4	77.3	4.5	72.8	95	-22.2
Yellow fever (1^st^ dose)	88.9	2.3	86.3	-1.3	87.8	0.2	87.2	-0.4	87.6	95	-7.4
Measles, mumps and rubella (1^st^ dose)	89.7	1.1	87.2	-1.4	91.2	2.6	87.4	-1.2	88.6	95	-6.4
Measles, mumps and rubella (2^nd^ dose)	86.9	5.2	78.1	-3.6	78.4	-3.3	80.5	-1.2	81.7	95	-13.3
Chickenpox (1^st^ dose)	78.1	-0.7	76.8	-2.0	80.3	1.5	80.3	1.5	78.8	95	-16.2
Hepatitis A	89.6	2.5	85.1	-2.0	87.6	0.5	85.8	-2.7	87.1	95	-7.9
Oral poliovirus vaccine (OPV)	84.9	3.3	78.4	-3.2	82.6	1.0	80.2	-1.4	81.6	95	-13.4
Diphtheria, pertussis and tetanus (DPT)	83.4	2.4	80.9	-0.1	79.5	-1.5	79.4	-0.6	81.0	95	-14.0

a) Difference.

We found that in Campo Grande no vaccine achieved coverage above 90%, in particular vaccines administered at birth: BCG (82.9%; dif -5.5) and hepatitis B (82.1%; dif -5.3). In Goiânia and Cuiabá, the second dose of the rotavirus vaccine had the poorest coverage (68.7%; dif -5.5 and 74.2%; dif -5.5). In Brasília, coverage of the third dose of pneumococcal vaccine (66.8%; dif -6.0) had the poorest performance (Table 3). 

## DISCUSSION

Full schedule vaccination coverage at 12 and 24 months old, with valid doses among children living in the state capitals of Midwest region of Brazil, was less than 80% and presented significant differences between the highest social strata in Campo Grande and Brasília, at 24 months. In Goiânia, vaccination coverage at 24 months was not significant, while in Cuiabá it was lower in stratum C, demonstrating the region’s heterogeneity. We found that vaccination coverage at 24 months reduced as income increased, except in Cuiabá, where the highest vaccination coverage was found in socioeconomic stratum A. Similar vaccination coverage heterogeneity was found by the ICV survey conducted in 2007 between social strata in 13 state capitals of the five Brazilian regions.^9,15^

The Midwest region has shown continuous development over the last few decades, with a growth rate of 1.23% per year, more than double the average of 0.52% for Brazil as a whole, with a high Human Development Index and high *per capita* Gross Domestic Product.^12^ Despite these favorable indicators, low vaccination coverage levels were found in all social strata in the region, with differences between the highest and lowest vaccination coverage per stratum in the same capital. For some authors, individuals from higher social strata fail to vaccinate or vaccinate their children due to vaccination hesitancy or recommendations made by health professionals.^15,16^ On the other hand, those belonging to lower social strata do not get vaccinated due to lack of access to health services and lack of knowledge that vaccines are important.^17,18^

Low vaccination coverage was found at 12 and 24 months old in all Midwest region state capitals, confirming the risk of resurgence of eliminated or controlled diseases and the threat to health services in Brazil. Understanding vaccination coverage in different regions contributes to the development of strategies that consider the specificities and needs of each location and is in line with the National Movement for Vaccination, the objective of which is to return to high vaccination coverage levels in Brazil.^19^ A study conducted carried out by Arroyo et al.,^20^ investigated areas with a drop in BCG, polio and MMR vaccination coverage in Brazil and also identified, like this study, a reduction in the number of people vaccinated in the Midwest region, although with a smaller drop than in the rest of Brazil. 

In general, lower vaccination coverage levels were found in those segments of the population with the best living conditions, a phenomenon different from that found for decades in Brazil in relation to vaccination coverage, whereby the population segments with poorer living conditions used to have lower vaccination coverage.^15,21,22^

Socioeconomic and intraregional differences, as well as differences in the characteristics of children, families and mothers can be seen between groups within socioeconomic strata and this can impact adherence to vaccination and, consequently, vaccination coverage.^11^

Considering the set of capital cities presented in this study, the highest vaccination coverage for the first 12 and 24 months of life was found in the city of Brasília. In turn, the lowest vaccination coverage was found in Campo Grande. Brasília is the capital of Brazil and the most populous city in the Midwest region, with better performance regarding HDI, Gini Index and *per capita* GDP indicators, in addition to having greater health service coverage.^12,13^ These characteristics may have contributed to its better performance regarding overall vaccination coverage and for most vaccines recommended for children under 24 months old. 

Valid vaccination coverage for the vaccination schedule recommended for the first 12 months of life, including doses of yellow fever vaccine, was better than at 24 months of age. The Brazilian Ministry of Health recommends that children have seven medical consultations in their first year of life (in the 1^st^ week, in the 1^st^ month, in the 2^nd^ month, in the 4^th^ month, in the 6^th^ month, in the 9^th^ month and in the 12^th^ month), and two consultations in their second year of life (in the 12^th^ and 15^th^ months). This provides the opportunity for children to be vaccinated at the time of medical consultations and consequently improves vaccination coverage performance in the first year of life.^23^ Notwithstanding, a reduction in vaccination coverage was found for those vaccines for which two or three doses are recommended, such as rotavirus, 5-in-1, meningococcal C and pneumococcal vaccine, administered in the first year of life.

Campo Grande had the poorest vaccination coverage at 24 months old age, and the poorest performance for each recommended vaccine. Low coverage of vaccines that should be administered at birth (BCG and hepatitis B) can be attributed to absence of vaccination rooms in that city’s maternity wards.^24^ The anti-vaccine infodemic, characterized by the wide dissemination of false information, with great potential to impact the population’s adherence to vaccination, especially after its significant increase during and after the COVID-19 pandemic,^25^ may also have contributed to the scenario of lower vaccination coverage among the Midwest region capital cities.

The results of this study need to be considered in light of its limitations, such as the demographic census not taking place in 2020, which obliged us to use old data to define the socioeconomic strata, which may have altered the comparisons in some cities where urban transformation has been more intense. The family level data used may help to identify these problems to a certain extent, given the limitations of the classification used.^11^ Collecting data during the COVID-19 pandemic also impacted response rates. Even so, it is noteworthy that the calculation of post-stratification sample weights took into account differences in responses between population groups and minimized such differences. The study’s strengths include its large sample size, in addition to the methodological rigor involved in collecting vaccination information.^11^ Taking photographs of vaccination cards with subsequent data entry by professionals with experience in the National Immunization Program enabled excellent quality of this information.

However, such limitations do not invalidate the results of this study, which point to the great heterogeneity that exists in vaccination coverage among children from different social strata living in the capital cities of the Midwest region of Brazil. Furthermore, differences were found between the highest and lowest vaccination coverage levels per stratum within the same capitals. It is also important to emphasize the low vaccination coverage levels found for the vast majority of vaccines recommended up to 24 months of life. Investigating factors intrinsic to economic and social variables can contribute to assertive intervention and, consequently, improve immunization indicators in the Midwest region of Brazil. Therefore, there is a need for targeted approaches, taking into consideration economic strata and vaccines with lower coverage. 
