# Supplementary material for: Vaccine coverage by social strata in state capitals in the Brazilian Midwest region: a household survey of children born in 2017 and 2018
Source: Epidemiol Serv Saude. 2024 Nov 1;33(spe2):e20231308. doi: 10.1590/S2237-96222024v33e20231308.especial2.en (PMC11529204; doi:10.1590/S2237-96222024v33e20231308.especial2.en)
Supplement: Translation

# Translation: Coberturas vacinais por estrato social nas capitais da região Centro-Oeste do Brasil: inquérito domiciliar em coorte de crianças nascidas em 2017 e 2018

**DOI:** 10.1590/S2237-96222024v33e20231308.especial2.pt

**Authors:** Jaqueline Costa Lima, Érica Marvila Garcia, Sandra Maria do Valle Leone de Oliveira, Wildo Navegantes de Araújo, Emmanuela Maria de Freitas Lopes, Sheila Araújo Teles, Karlla Antonieta Amorim Caetano, Ana Izabel Passarela Teixeira, Bárbara Manuella Cardoso Sodré Alves, Ana Paula França, José Cássio de Moraes, Carla Magda Allan Santos Domingues, Grupo ICV 2020, Adriana Ilha da Silva, Alberto Novaes Ramos, Ana Paula França, Andrea de Nazaré Marvão Oliveira, Antonio Fernando Boing, Carla Magda Allan Santos Domingues, Consuelo Silva de Oliveira, Ethel Leonor Noia Maciel, Ione Aquemi Guibu, Isabelle Ribeiro Barbosa Mirabal, Jaqueline Caracas Barbosa, Jaqueline Costa Lima, José Cássio de Moraes, Karin Regina Luhm, Karlla Antonieta Amorim Caetano, Luisa Helena de Oliveira Lima, Maria Bernadete de Cerqueira Antunes, Maria da Gloria Teixeira, Maria Denise de Castro Teixeira, Maria Fernanda de Sousa Oliveira Borges, Rejane Christine de Sousa Queiroz, Ricardo Queiroz Gurgel, Rita Barradas Barata, Roberta Nogueira Calandrini de Azevedo, Sandra Maria do Valle Leone de Oliveira, Sheila Araújo Teles, Silvana Granado Nogueira da Gama, Sotero Serrate Mengue, Taynãna César Simões, Valdir Nascimento, Wildo Navegantes de Araújo

**Affiliations:** 1 Universidade Federal de Mato Grosso, Faculdade de Enfermagem, Cuiabá, MT, Brasil; 2 Secretaria de Saúde de Marataízes, Vigilância em Saúde, Espírito Santo, ES, Brasil; 3 Fundação Oswaldo Cruz, Campo Grande, MS, Brasil; 4 Universidade de Brasília, Faculdade de Ceilândia, Brasília, DF, Brasil; 5 Secretaria Municipal de Saúde, Programa de Pós-graduação em Doenças Infecciosas e Parasitárias, Campo Grande, MS, Brasil; 6 Universidade Federal de Goiás, Goiânia, GO, Brasil; 7 Universidade Federal de Mato Grosso do Sul, Paranaíba, MS, Brasil; 8 Laboratório de Estudos Farmacêuticos, Universidade de Brasília, Brasília, DF, Brasil; 9 Faculdade de Ciências Médicas da Santa Casa de São Paulo, Departamento de Saúde Coletiva, São Paulo, SP, Brasil; 10 ﻿Organização Pan-Americana da Saúde, Brasília, DF, Brasil; Universidade Federal do Espírito Santo, Vitória, ES, Brasil; Universidade Federal do Ceará, Departamento de Saúde Comunitária, Fortaleza, CE, Brasil; Faculdade Ciências Médicas Santa Casa de São Paulo, São Paulo, SP, Brasil; Secretaria de Estado da Saúde do Amapá, Macapá, AP, Brasil; Universidade Federal de Santa Catarina, SC, Brasil; Organização Pan-Americana da Saúde, Brasília, DF, Brasil; Instituto Evandro Chagas, Belém, PA, Brasil; Faculdade de Ciências Médicas Santa Casa de São Paulo, Departamento de Saúde Coletiva, São Paulo, SP, Brasil; Universidade Federal de Mato Grosso, Cuiabá, MT, Brasil; Universidade Federal do Paraná, Curitiba, PR, Brasil; Universidade Federal de Goiás, Goiânia, GO, Brasil; Universidade Federal do Piauí, Teresina, PI, Brasil; Universidade de Pernambuco, Faculdade de Ciências Médicas, Pernambuco, PE, Brasil; Instituto de Saúde Coletiva, Universidade Federal da Bahia, Salvador, BA, Brasil; Secretaria de Estado da Saúde de Alagoas, Maceió, AL, Brasil; Universidade Federal do Acre, Rio Branco, AC, Brasil; Universidade Federal do Maranhão, Departamento de Saúde Pública, São Luís, MA, Brasil; Universidade Federal de Sergipe, Aracaju, SE, Brasil; Secretaria Municipal de Saúde, Boa Vista, RR, Brasil; Fundação Oswaldo Cruz, Mato Grosso do Sul, Campo Grande, MS, Brasil; Fundação Oswaldo Cruz, Escola Nacional de Saúde Pública Sergio Arouca, Rio de Janeiro, RJ, Brasil; Universidade Federal do Rio Grande do Sul, Porto Alegre, RS, Brasil; Fundação Oswaldo Cruz, Instituto de Pesquisa René Rachou, Belo Horizonte, MG, Brasil; Secretaria de Desenvolvimento Ambiental de Rondônia, Porto Velho, RO, Brasil; Universidade de Brasília, Brasília, DF, Brasil

**Keywords:** Programas de Imunização, Cobertura Vacinal, Fatores Socioeconômicos, Desigualdades Sociais em Saúde, Inquéritos Populacionais

## Abstract

**Objetivo:**

Analisar a cobertura vacinal completa em nascidos vivos em 2017 e 2018, nas capitais da região Centro-Oeste do Brasil, segundo estratos sociais.

**Métodos:**

Inquérito domiciliar de base populacional com amostragem por conglomerados. Analisou-se a cobertura vacinal completa em crianças aos 12 e 24 meses de idade e os fatores sociodemográficos.

**Resultados:**

Foram analisadas 5.715 crianças. A cobertura completa aos 12 meses de idade foi 67,9% (IC_95_% 65,4;70,4) e aos 24 meses de idade foi 48,2% (IC_95_% 45,3;51,1). A maior cobertura foi da vacina pneumococo (91,3%) e a pior da segunda dose da vacina rotavírus (74,2%). Em Campo Grande, nenhuma vacina alcançou cobertura acima de 90%, destacando-se as vacinas BCG (82,9%) e hepatite B (82,1%). Campo Grande e Brasília tiveram piores coberturas vacinais no estrato social alto (24 meses de idade).

**Conclusão:**

A cobertura vacinal na região Centro-Oeste foi inferior a 80%, abaixo da meta preconizada e associada com fatores socioeconômicos.

## Contribuições do estudo

**Principais resultados:**

As coberturas vacinais aos 24 meses de idade, nas capitais do Centro-Oeste, variaram de 39,9%, em Campo Grande, a 54,5% em Brasília, todas abaixo da meta preconizada pelo Programa Nacional de Imunizações.

**Implicações para os serviços:**

Os resultados encontrados apontam para a urgência no planejamento de ações que visem melhorar a cobertura vacinal com abordagens direcionadas, considerando os estratos sociais e as vacinas com menor cobertura vacinal.

**Perspectivas:**

Pesquisas futuras que investiguem a heterogeneidade das coberturas vacinais na região Centro-Oeste podem auxiliar no entendimento das baixas coberturas encontradas para a maioria das vacinas preconizadas, principalmente até os 24 meses de vida.

## INTRODUÇÃO

A redução da cobertura vacinal tem sido observada em âmbito mundial, na última década.^1^ Apesar da existência de um Plano de Ação Global para Vacinas, proposto pela Organização Mundial da Saúde em 2022, cerca de 14 milhões de crianças não completaram o esquema de vacinação, pois viviam em países de média e baixa rendas, como Angola, Brasil, República Democrática do Congo, Etiópia, Índia, Indonésia, Moçambique, Nigéria, Paquistão e Filipinas.^2^

No Brasil, a queda da cobertura vacinal teve início em 2012, com maior gravidade durante a pandemia de covid-19.^3^ Entre os anos de 2019 e 2021, para as vacinas contra difteria, tétano e pertússis (DTP), sarampo e pneumocócica,^3^ não foi alcançada a cobertura vacinal desejada de 90%, um importante indicador de monitoramento global definido pela Agenda de Imunização 2030.^4^ A causa dessa redução nas coberturas vacinais é multifacetada e requer a compreensão das intervenções em saúde pública no país, a operacionalidade das ações, como também a conjuntura geográfica e política regional, nacional e internacional.^5^

A história da implementação das vacinas no Brasil data do início do século XIX, em que se observa a introdução gradual e gratuita de imunobiológicos. A criação do Programa Nacional de Imunizações (PNI), em 1973, foi um marco para a saúde pública do Brasil e mudou o cenário epidemiológico das infecções transmissíveis em todo o país. A partir da introdução do programa foram iniciadas as ações sistematizadas de erradicação de doenças imunopreveníveis, com a expansão da oferta de vacinas para toda a população brasileira.^5,6^

Apesar das significativas reduções nas disparidades sociais e melhora nos indicadores de saúde ao longo das últimas décadas no Brasil, dados administrativos apresentam diferenças intrarregionais e inter-regionais nas coberturas vacinais, em diversas regiões do país. Por outro lado, validar esses resultados, por meio da realização de inquéritos domiciliares, é desejável, uma vez que estudos anteriores mostraram grandes discrepâncias entre dados administrativos e aqueles oriundos de inquéritos.^7,8^

Assim, passados mais de dez anos após o último inquérito domiciliar de coberturas vacinais realizado nas capitais do Brasil,^9^ torna-se necessário entender o panorama atual dessas coberturas na região Centro-Oeste do país, uma região estratégica, porém carente de produção científica.^10^ Acreditamos que a validação desses dados permitirá identificar possíveis oportunidades de melhorias nos indicadores de cobertura vacinal, gerar hipóteses para novas pesquisas e ampliar o conhecimento acerca dos fatores que podem estar relacionados à vacinação. Assim, este estudo tem como objetivo analisar as coberturas vacinais em nascidos vivos em 2017 e 2018, nas capitais da região Centro-Oeste do Brasil, segundo estrato social. 

## MÉTODOS

### Desenho do estudo

Trata-se de um inquérito de base populacional, com dados do Inquérito de Cobertura Vacinal (ICV), que avaliou as coberturas vacinais por doses válidas na região Centro-Oeste do Brasil. O ICV foi realizado nos 26 estados e no Distrito Federal, entre setembro de 2020 e março de 2022.^11^

### Contexto

A região Centro-Oeste é a segunda maior região em extensão territorial, e possui população estimada de 16,3 milhões de habitantes e densidade demográfica que varia, nas capitais, de 97,22 hab./km^2^ em Campo Grande, a 1.776,8 hab./km^2^ em Goiânia (Quadro 1).^12^ Sua economia é baseada na agricultura, pecuária e extração mineral, com crescimento significativo em diversos setores.^13^

**Quadro 1:** Descrição das características socioeconômicas e demográficas das quatro capitais da região Centro-Oeste do Brasil

**Características sociodemográficas**	**Cuiabá**	**Campo Grande**	**Goiânia**	**Distrito Federal**
População^a^	650.912	897.938	1.437.237	2.817.068
População ≤ 4 anos^a^	43.647	59.766	83.676	166.848
Indice de desenvolvimento humano^a^	0,785 (muito alto)	0,784 (alto)	0,799 (muito alto)	0,824 (muito alto)
Índice de Gini^b^	0,5293	0,5070	0,4751	0,6370
Produto interno bruto *per capita* ^a^	42.918,31	33.243,63	33.826,84	87.016,16
Densidade demográfica^a^	150,41	111,09	1.970,72	489,01
Taxa de mortalidade infantil^a^	12,92	10,29	9,26	9,76
Índice de Vulnerabilidade Social	0,261	0,27	0,291	0,294

a) Censo de 2022; b) Censo de 2010.

### Participantes

A população de estudo foi composta por crianças nascidas em 2017 e 2018, e residentes em área urbana das capitais Campo Grande, Cuiabá e Goiânia, e do Distrito Federal. 

### Variáveis

As variáveis de estudo foram as coberturas vacinais válidas completas e incompletas aos 12 e 24 meses de idade, e as características socioeconômicas e demográficas, como: estrato social (A – alto, B – médio, C – baixo, D – muito baixo) –, definido de acordo com dados de renda e escolaridade do chefe da família;^12^ nível de consumo familiar (alto, médio, baixo, muito baixo e não respondeu) –, definido segundo os pontos de corte: alto (42 pontos e mais), médio (27 a 41 pontos), baixo (16 a 26 pontos) e muito baixo (< 16 pontos);^14^ aglomeração familiar (presença de mais de três moradores por cômodo usado como dormitório); renda familiar mensal (até R$ 1.000,00, de R$ 1001,00 a R$ 3.000,00, de R$ 3.001,00 a R$ 8.000,00, acima de R$ 8.000,00 e não respondeu); percentual de avós que residem no domicílio; características maternas: escolaridade em anos de estudo (até 8 anos, 9 - 2 anos, 13 - 15 anos, 16 anos ou mais, não sabe ou não quis responder); faixa etária (< 20 anos, 20 - 34 anos, 35 anos ou mais, não sabe ou não quis responder); raça/cor da pele autodeclarada (branca, preta, parda, amarela, indígena, não sabe/não quis responder); percentual de mães com trabalho remunerado, percentual de mães que possuem companheiro; número de filhos (1 - 3 filhos, 4 - 7 filhos e > 7 filhos); características das crianças: sexo (feminino e masculino); ordem de nascimento (primeiro, segundo, terceiro, quarto ou mais, não respondeu); raça/cor da pele (branca, preta, parda, amarela, indígena, não sabe/não quis responder) e percentual de crianças que frequentam creche. 

Para as análises de cobertura vacinal, foram utilizadas as seguintes definições, de acordo com Barata et al. (2023):^11^

- Dose válida: cumprimento do calendário proposto, levando-se em conta as idades preconizadas pelo calendário oficial do PNI e os intervalos corretos entre as doses.
- Cobertura vacinal completa para os primeiros 12 meses de vida (“esquema básico”), que consiste nas vacinas: bacilo de Calmette-Guérin (BCG), hepatite B, três doses de vacina pentavalente (DTP + *Hemophilus influenzae* tipo B + hepatite B) e de vacina inativada de poliovírus (VIP), duas doses de vacina contra rotavírus, duas doses de vacina contra meningococo C e pneumococo, e uma dose de vacina contra febre amarela.
- Cobertura vacinal completa aos 24 meses, que incluía, além das vacinas do esquema básico, duas doses de tríplice viral (sarampo, rubéola e caxumba), uma dose de hepatite A, de varicela e da vacina oral poliomielite Bivalente (VOPb); e uma dose de reforço com DTP (difteria, tétano e pertússis), meningococo C, pneumococo.
- Para o cálculo da cobertura vacinal, foi necessário fazer a junção de vários imunobiológicos, pois alguns são aplicados apenas em serviços privados, também incluídos na presente pesquisa.^11^ As junções das vacinas foram assim descritas: pentavalente (pentavalente, hexavalente e acelular (Acel)); VIP (VIP e hexavalente); meningocócica C (meningocócica C e meningocócica ACWY); tríplice viral (tríplice viral e tetravalente); varicela (varicela e tetravalente); dose de reforço contra a poliomielite (VOP, doses aplicadas acima de 1 ano da VIP ou hexavalente ‒ doses não utilizadas no esquema básico da VIP); 1º reforço da vacina DTP (DTP, doses aplicadas acima de 1 ano das vacinas pentavalente, hexavalente ou Acel não utilizadas no esquema básico da vacina pentavalente).

### Fonte de dados

As fontes de dados utilizadas foram o questionário elaborado para a realização do ICV, contendo as variáveis socioeconômicas e demográficas já descritas, e a foto da caderneta de vacinação da criança, contendo a informação sobre as vacinas aplicadas.^11^

### Amostra

Foi realizada uma amostra complexa, definida previamente, dependendo do número de nascidos vivos registrados no Sistema de Informações sobre Nascidos Vivos, em 2017 e 2018, cujos pesos amostrais foram calculados para cada domicílio entrevistado. Inicialmente foram obtidos pesos amostrais básicos (inverso das probabilidades de inclusão dos domicílios entrevistados), e em seguida esses pesos foram calibrados para totais populacionais conhecidos. Em cada cidade da região Centro-Oeste, foram realizados de dois a quatro inquéritos, sendo dois em Cuiabá, três em Campo Grande e quatro em Goiânia e Brasília. Foram consideradas perdas as recusas, impossibilidade de realização da entrevista após três tentativas em horários e dias diferentes e impossibilidade de localizar o número previsto de crianças após busca ativa em toda a área dos conglomerados sorteados. ^11^ Os procedimentos operacionais, o cálculo amostral e demais informações técnicas do ICV estão descritas em Barata et al., 2023.^11^

### Análise estatística

Foram calculadas as coberturas vacinais (doses válidas), tomando como numerador as crianças que receberam todas as vacinas recomendadas no primeiro ano de vida (incluída a vacina de febre amarela) e como denominador as crianças nascidas em 2017 e 2018 incluídas no estudo e multiplicadas por 100 para as capitais do Centro-Oeste, aos 12 e aos 24 meses de idade, por estrato social. Para o cálculo da cobertura vacinal, foi considerado um intervalo de confiança de 95% (IC_95_%). O teste qui-quadrado foi utilizado para avaliar diferença entre as coberturas vacinais nas capitais da região Centro-Oeste, aos 12 e 24 meses de idade, por estrato social. Valores de p 0,05 foram considerados estatisticamente significativos. A diferença entre as coberturas vacinais de cada vacina foi estimada subtraindo-se a cobertura vacinal da região Centro-Oeste da cobertura vacinal de cada capital da região (denominada de dif). O *software* Stata versão 17, módulo *survey data analysis*, foi utilizado para a análise dos dados. 

### Aspectos éticos

Este estudo foi aprovado pelos Comitês de Ética em Pesquisa com Seres Humanos do Instituto de Saúde Coletiva da Universidade Federal da Bahia, sob parecer nº 3.366.818, em 4 de junho de 2019, com Certificado de Apresentação de Apreciação Ética (CAAE) 4306919.5.0000.5030; e da Irmandade da Santa Casa de São Paulo, sob parecer nº 4.380.019, em 4 de novembro de 2020, com CAAE 39412020.0.0000.5479. Todos os entrevistados assinaram o termo de consentimento livre e esclarecido para a realização da entrevista e a autorização para que a caderneta de vacinação fosse fotografada. 

## RESULTADOS

Foram incluídas neste estudo 5.715 crianças, sendo 31,7% (1.811/5.715) de Goiânia, 31,6% (1.809/5.715) de Brasília, 22,4% (1.281/5.715) de Campo Grande e 14,3% (814/5.715) de Cuiabá. Do total, 22,7% (1.297) pertencem ao estrato socioeconômico A, 25,4% (1.451) ao estrato B, 25,9% (1.480) ao estrato C e 26,0% (1.487) ao estrato D. Das capitais estudadas, ocorreram perdas no município de Cuiabá (9,8%) e em Campo Grande (5,4%). 

As características sociodemográficas das famílias, mães e crianças participantes incluídas neste estudo estão apresentadas na Tabela 1. 

Todas as coberturas vacinais para os primeiros 12 e 24 meses de vida, em cada uma das cinco cidades participantes e por estrato social, estão apresentadas na Tabela 2. 

**Tabela 1:** Características sociodemográficas das famílias, mães e crianças participantes das quatro capitais da região Centro-Oeste, Brasil, 2020-2022

	**Campo Grande N (%)**	**Cuiabá N (%)**	**Goiânia N (%)**	**Brasília N (%)**
Número de famílias incluídas	1.281	814	1.811	1.809
**Estrato social**				
A	271 (20,4)	131 (21,6)	445 (10,9)	450 (8,6)
B	324 (12,9)	226 (13,7)	447 (14,5)	454 (10,6)
C	343 (9,4)	230 (17,4)	452 (20,7)	455 (31,0)
D	343 (57,3)	227 (47,3)	467 (53,9)	450 (49,8)
**Nível de consumo familiar**				
Alto	61 (5,1)	15 (2,2)	37 (1,8)	286 (8,0)
Médio	431 (23,7)	145 (24,1)	509 (16,0)	765 (31,4)
Baixo	422 (31,2)	342 (33,9)	770 (51,5)	441 (32,3)
Muito baixo	324 (35,0)	295 (37,4)	445 (28,1)	244 (24,6)
Não respondeu	43 (5,0)	17 (2,4)	50 (2,6)	73 (3,7)
Aglomeração familiar	87 (7,5)	71 (10,2)	59 (6,9)	84 (9,0)
**Renda familiar mensal**				
Até R$ 1.000,00	156 (20,1)	196 (23,7)	85 (12,1)	205 (19,6)
R$ 1.001,00 - R$ 3.000,00	419 (34,6)	297 (34,6)	631 (41,1)	368 (30,3)
R$ 3.001,00 - R$ 8.000,00	404 (25,0)	147 (26,8)	741 (33,1)	370 (21,4)
Acima de R$ 8.000,00	172 (10,5)	57 (6,0)	165 (7,1)	689 (22,1)
Não respondeu	130 (9,8)	117 (8,9)	189 (6,6)	177 (6,6)
Avó mora junto	327 (29,2)	231 (24,5)	322 (21,1)	449 (31,1)
**Características maternas**				
**Anos de estudo**				
Até 8 anos	85 (10,0)	36 (5,0)	84 (4,5)	60 (4,7)
9 - 12 anos	152 (16,2)	137 (18,5)	215 (13,)	121 (12,3)
13 - 15 anos	435 (35,2)	367 (42,4)	820 (47,7)	483 (37,5)
16 ou mais	572 (34,0)	257 (31,7)	642 (31,4)	1091 (42,2)
Não sabe ou não quis responder	37 (4,6)	17 (2,4)	50 (2,6)	54 (3,3)
**Faixa etária (anos)**				
< 20	35 (4,5)	22 (3,9)	33 (4,9)	20 (3,2)
20 - 34	689 (55,9)	516 (63,1)	1.172 (68,5)	787 (53,0)
35 ou mais	553 (39,3)	275 (32,8)	583 (25,4)	997 (43,7)
Não sabe ou não quis responder	4 (0,3)	1 (0,2)	23 (1,2)	5 (0,1)
**Raça/cor da pele autodeclarada da mãe**				
Branca	690 (46,6)	163 (18,1)	684 (39,6)	838 (36,1)
Preta	55 (7,0)	154 (22,8)	117 (8,0)	141 (10,7)
Parda	465 (39,5)	473 (55,8)	949 (48,7)	746 (49,7)
Amarela	27 (1,6)	8 (0,5)	13 (1,4)	25 (0,8)
Indígena	6 (0,8)	4 (0,9)	2 (0,4)	6 (0,4)
Não sabe ou não quis responder	38 (4,5)	12 (1,9)	46 (1,9)	53 (2,3)
**Trabalho remunerado**	779 (56,0)	429 (56,7)	1.087 (52,4)	1.158 (59,2)
**Tem companheiro**	994 (74,2)	618 (82,5)	1.471 (81,8)	1.457 (75,7)
**Número de filhos**				
1 - 3 filhos	1.159 (87,3)	700 (82,3)	1.650 (89,4)	1.690 (91,3)
4 - 7 filhos	120 (12,6)	110 (17,6)	151 (10,4)	112 (8,3)
> 7 filhos	1 (0,1)	4 (0,1)	8 (0,2)	2 (0,4)
**Características das crianças**				
**Sexo**				
Masculino	651 (53,6)	424 (50,3)	891 (48,4)	909 (50,8)
Feminino	630 (46,4)	390 (49,7)	920 (51,6)	900 (49,2)
**Ordem de nascimento**				
Primeiro	576 (38,4)	337 (37,9)	841 (44,1)	891 (49,3)
Segundo	441 (35,0)	249 (33,5)	633 (35,3)	621 (30,1)
Terceiro	169 (16,4)	139 (15,6)	212 (13,3)	210 (14,4)
Quarto ou mais	94 (10,1)	89 (13,0)	124 (6,7)	83 (6,0)
Não respondeu	1 (0,02)	0 (0,0)	1 (0,6)	4 (0,2)
**Raça/cor da pele da criança**				
Branca	834 (59,8)	257 (28,2)	837 (46,9)	1.012 (45,5)
Preta	27 (4,1)	91 (11,2)	57 (4,9)	80 (6,4)
Parda	396 (34,0)	457 (57,3)	905 (47,0)	687 (47,0)
Amarela	16 (1,3)	8 (3,2)	10 (1,1)	16 (0,6)
Indígena	7 (0,8)	1 (0,04)	1 (0,06)	2 (0,2)
Não sabe ou não quis responder	1 (0,01)	0 (0,0)	1 (0,0)	12 (0,3)
**Frequenta creche**	617 (46,0)	372 (45,2)	678 (31,1)	870 (39,8)

**Tabela 2:** Coberturas vacinais para os primeiros 12 e 24 meses de vida, segundo estrato social, das quatro capitais da região Centro-Oeste do Brasil, 2020-2022

	Cobertura vacinal aos 12 meses (%) (IC_95_ **%**)^a^	p-valor^b^	Cobertura vacinal aos 24 meses (%) (IC_95_ **%**)^a^	p-valor^b^
**Campo Grande**	**60,7 (54,6;66,5)**		**39,9 (35,0;45,1)**	
**A**	58,4 (44,6;70,9)		25,0 (17,5;34,6)	
**B**	78,1 (65,7;86,8)	0,076	48,3 (38,0;58,7)	0,002
**C**	59,3 (47,9;69,8)		40,4 (31,5;49,2)	
**D**	57,9 (49,6;65,7)		43,4 (36,6;50,4)	
**Cuiabá**	**60,4 (54,3;66,3)**		**46,2 (39,4;53,2)**	
**A**	72,4 (59,4;82,5)		55,9 (42,1;68,9)	
**B**	50,3 (35,7;64,8)	0,055	47,1 (39,1;55,2)	0,050
**C**	53,6 (42,3;64,6		31,8 (23,5;41,5)	
**D**	62,1 (56,2;67,6)		46,8 (37,3;56,5)	
**Goiânia**	**62,1 (57,3;66,6)**		**47,2 (40,8;53,8)**	
**A**	64,7 (44,9;80,5)		43,2 (22,9;66,1)	
**B**	58,2 (51,8;64,4)	0,805	37,1 (29,3;45,6)	0,427
**C**	60,9 (53,6;67,8)		45,8 (41,0;50,8)	
**D**	63,0 (55,7;69,8)		51,3 (40,8;53,8)	
**Brasília**	**76,3 (72,5;79,8)**		**54,5 (49,8;59,1)**	
**A**	75,9 (70,0;80,8)		28,2 (22,7;34,3)	
**B**	73,6 (65,9;80,1)	0,238	29,8 (22,9;37,9)	< 0,001
**C**	80,7 (73,8;86,2)		59,4 (49,5;68,6)	
**D**	74,2 (67,8;79,7)		61,1 (54,3;67,5)	

a) (IC_95_%): Intervalo de confiança de 95%; b) Teste qui-quadrado de Pearson.

A cobertura vacinal válida das capitais da região Centro-Oeste para o esquema vacinal recomendado para os primeiros 12 meses de vida, incluindo as doses da vacina de febre amarela, foi de 67,9% (IC_95_% 65,4;70,4). Analisando-se por cidades participantes, a maior cobertura vacinal foi observada na cidade de Brasília (76,3%) (IC_95_% 72,5;79,8), e a menor cobertura em Cuiabá (60,4%) (IC_95_% 54,3;66,3). Ao contrário do que foi observado aos 24 meses, não se observou diferença estatística na cobertura vacinal entre os estratos sociais. 

A cobertura vacinal válida das capitais do Centro-Oeste para o esquema vacinal recomendado para 24 meses, incluindo as doses da vacina de febre amarela, foi de 48,2% (IC_95_% 45,3;51,1). Analisando-se por cidades participantes, a maior cobertura vacinal para os primeiros 24 meses de vida foi observada na cidade de Brasília (54,5%) (IC_95_% 49,8;59,1), enquanto a menor cobertura vacinal foi observada em Campo Grande (39,9%) (IC_95_% 35,0;45,1) (Tabela 2). 

Ao considerarmos os estratos sociais, observou-se diferença estatística na cobertura vacinal aos 24 meses para Campo Grande e Brasília. Em Campo Grande e Brasília, menores coberturas vacinais foram encontradas no estrato A. Já em Cuiabá e Goiânia, as piores coberturas vacinais foram observadas nos estratos C (31,8%) (IC_95_% 23,5;41,5) e B (37,1%) (IC_95_% 29,3;45,6), respectivamente, embora não tenha havido significância estatística por estrato social em Goiânia (Tabela 2).

A cobertura vacinal de cada uma das vacinas está apresentada na Tabela 3. A maior cobertura foi observada para a primeira dose da vacina pneumococo (91,3%) e a menor para a segunda dose da vacina rotavírus (74,2%). Foi comparada a cobertura vacinal no conjunto das capitais do Centro-Oeste em relação às capitais. 

**Tabela 3:** Coberturas vacinais atualizadas por esquema de vacinas e as diferenças entre as coberturas nas capitais e a cobertura na região Centro-Oeste do Brasil, 2020-2022

Vacinas	Cidades								Centro-Oeste	Meta PNI	Dif^a^
	Brasília		Campo Grande		Cuiabá		Goiânia				
	Cobertura vacinal (%)	Dif^a^	Cobertura vacinal (%)	Dif^a^	Cobertura vacinal (%)	Dif^a^	Cobertura vacinal (%)	Dif^a^	Cobertura vacinal (%)	Cobertura vacinal (%)	
BCG	90,9	2,5	82,9	-5,5	90,0	1,6	89,1	0,7	88,4	90	-1,6
Hepatite B ao nascer	89,9	2,5	82,1	-5,3	88,9	1,5	87,9	0,5	87,4	95	-7,6
Pentavalente (1^a^ dose)	90,6	-0,1	88,8	-1,9	92,5	1,8	91,4	0,7	90,7	95	-4,3
Pentavalente (2^nd^ dose)	90,1	1,0	86,2	-2,9	90,4	1,3	89,5	0,4	89,1	95	-5,9
Pentavalente (3^rd^ dose)	88,8	2,6	82,5	-3,7	86,1	-0,1	86,3	0,1	86,2	95	-8,8
Vip (1^st^ dose)	91,2	0,3	89,7	-1,2	91,8	0,9	91,1	0,2	90,9	95	-4,1
Vip (2^nd^ dose)	90,4	0,9	87,6	-1,9	90,5	1,0	89,5	0,0	89,5	95	-5,5
Vip 3 (3^rd^ dose)	89,1	2,0	84,3	-2,8	81,1	-6,0	87,0	-0,1	87,1	95	-7,9
Rotavírus (1^st^ dose)	88,5	1,6	85,9	-1,0	87,6	0,7	85,8	-1,1	86,9	95	-8,1
Rotavírus (2^nd^ dose)	80,4	6,2	74,6	0,4	72,2	-2,0	68,7	-5,5	74,2	90	-15,8
Menigocócica C (1^a^ dose)	91,4	0,3	89,6	-1,5	93,2	2,1	91,0	-0,1	91,1	95	-4,4
Menigocócica C (2^nd^ dose)	89,6	1,2	86,6	-1,8	88,0	-0,4	88,7	0,3	88,4	95	-6,6
Menigocócica C (3^rd^ dose)	77,5	0,4	72,8	-4,4	78,9	1,8	79,0	1,9	77,1	95	-17,9
Pneumococo (1^st^ dose)	91,4	0,1	89,9	-1,4	93,2	1,9	91,2	-0,1	91,3	95	-3,7
Pneumococo (2^nd^ dose)	90,8	1,0	88,1	-1,7	90,5	0,7	89,7	-0,1	89,8	95	-5,2
Pneumococo (3^rd^ dose)	66,8	-6,0	69,4	-3,4	81,2	8,4	77,3	4,5	72,8	95	-22,2
Febre amarela (1^st^ dose)	88,9	2,3	86,3	-1,3	87,8	0,2	87,2	-0,4	87,6	95	-7,4
Triplíce viral (1^st^ dose)	89,7	1,1	87,2	-1,4	91,2	2,6	87,4	-1,2	88,6	95	-6,4
Triplíce viral (2^nd^ dose)	86,9	5,2	78,1	-3,6	78,4	-3,3	80,5	-1,2	81,7	95	-13,3
Varicela (1^st^ dose)	78,1	-0,7	76,8	-2,0	80,3	1,5	80,3	1,5	78,8	95	-16,2
Hepatite A	89,6	2,5	85,1	-2,0	87,6	0,5	85,8	-2,7	87,1	95	-7,9
Vacina oral poliomielite (VOP)	84,9	3,3	78,4	-3,2	82,6	1,0	80,2	-1,4	81,6	95	-13,4
Vacina adsorvida difteria, tétano e pertússis DTP	83,4	2,4	80,9	-0,1	79,5	-1,5	79,4	-0,6	81,0	95	-14,0

a) Diferença.

Observou-se que em Campo Grande nenhuma vacina alcançou cobertura acima de 90%, destacando-se as vacinas aplicadas ao nascer: vacinas BCG (82,9%; dif -5,5) e hepatite B (82,1%; dif -5,3). Em Goiânia e Cuiabá, as piores coberturas foram da segunda dose da vacina rotavírus (68,7%; dif -5,5 e 74,2%; dif -5,5). Em Brasília, a cobertura da terceira dose da vacina pneumococo (66,8%; dif -6,0) foi a de pior desempenho (Tabela 3). 

## DISCUSSÃO 

A cobertura vacinal do esquema completo aos 12 e aos 24 meses de idade, com doses válidas em crianças residentes nas capitais da região Centro-Oeste do país, foi inferior a 80% e apresenta diferenças significantes entre os estratos sociais mais altos em Campo Grande e Brasília, aos 24 meses. Em Goiânia, a cobertura vacinal aos 24 meses não foi significativa, e em Cuiabá foi menor no estrato C, demonstrando a heterogeneidade da região. Observou-se que, à medida que aumenta a renda, reduzem-se as coberturas vacinais aos 24 meses, exceto para a capital Cuiabá, cuja cobertura vacinal maior foi no estrato socioeconômico A. Tal heterogeneidade nas coberturas vacinais foi observada entre estratos sociais em 13 capitais das cinco regiões brasileiras no ICV realizado em 2007.^9,15^

A região Centro-Oeste tem apresentado um desenvolvimento contínuo ao longo das últimas décadas, com uma taxa de crescimento de 1,23% ao ano, mais que o dobro da média do país de 0,52%, com alto índice de desenvolvimento humano (IDH) e produto interno bruto (PIB) *per capita*.^12^ Apesar desses indicadores favoráveis, observaram-se baixas coberturas vacinais em todos os estratos sociais da região, com diferenças entre as maiores e menores coberturas por estrato na mesma capital. Para alguns autores, indivíduos de estratos sociais mais elevados deixam de vacinar ou vacinam seus filhos por hesitação vacinal ou recomendações de profissionais de saúde.^15,16^ Já os pertencentes a estratos sociais mais baixos não se vacinam por falta de acesso aos serviços de saúde e desconhecimento de que as vacinas são importantes.^17,18^

Uma baixa cobertura vacinal foi observada aos 12 e aos 24 meses de idade em todas as capitais do Centro-Oeste, ratificando o risco de ressurgimento de doenças eliminadas ou controladas e o retrocesso sanitário do país. Entender as coberturas vacinais nas diversas regiões contribui para a elaboração de estratégias que considerem as especificidades e necessidades de cada localidade e vai ao encontro do Movimento Nacional pela Vacinação, cujo objetivo é retomar as altas coberturas vacinais no país.^19^ Estudo realizado por Arroyo et al.^20^ investigou as áreas com queda da cobertura de BCG, poliomielite e tríplice viral no Brasil e também identificou, assim como este estudo, uma redução no número de vacinados na região Centro-oeste, com uma queda menor do que no restante do país. 

De modo geral, observaram-se menores coberturas vacinais naquelas camadas da população que apresentam as melhores condições de vida, fenômeno diferente daquele encontrado por décadas no país em relação às coberturas ,em que as parcelas populacionais com piores condições de vida possuíam menores coberturas vacinais.^15,21,22^

 Diferenças socioeconômicas, intrarregionais e nas características das crianças, famílias e da mãe são observadas entre os grupos pertencentes aos estratos socioeconômicos, e isso pode impactar a adesão à vacina e, consequentemente, à cobertura vacinal.^11^

Considerando-se o conjunto de capitais apresentado neste estudo, a maior cobertura vacinal para os primeiros 12 e 24 meses de vida foi observada na cidade de Brasília. Por sua vez, a menor cobertura vacinal foi observada em Campo Grande. Brasília é a capital do país e a cidade mais populosa do Centro-Oeste, com melhor desempenho nos indicadores IDH, Índice de Gini e PIB *per capita*, além de maior cobertura de serviços de saúde.^12,13^ Essas características podem ter contribuído para o melhor desempenho na cobertura vacinal global e na maioria das vacinas recomendadas para crianças com menos de 24 meses de idade. 

A cobertura vacinal válida para o esquema vacinal recomendado para os primeiros 12 meses de vida, incluídas as doses da vacina contra febre amarela, apresentou um desempenho melhor do que aos 24 meses de idade. No primeiro ano de vida, o Ministério da Saúde recomenda sete consultas médicas para a criança (na 1ª semana, no 1º mês, no 2º mês, no 4º mês, no 6º mês, no 9º mês e no 12º mês) e no segundo ano de vida duas consultas (no 12º e no 15º mês). Isso oportuniza a vacinação da criança no período da consulta e consequentemente melhora o desempenho da cobertura vacinal no primeiro ano de vida.^23^ No entanto, observou-se uma redução da cobertura vacinal para aquelas vacinas com duas ou três doses, como a rotavírus, pentavalente, meningocócica C e pneumocócica, administradas ainda no primeiro ano de vida.

Campo Grande apresentou a pior cobertura vacinal aos 24 meses de idade, e o pior desempenho para cada vacina recomendada. As baixas coberturas para as vacinas que devem ser aplicadas ao nascimento (BCG e hepatite B) podem ser atribuídas à ausência de salas de vacinas nas maternidades da cidade.^24^ A infodemia antivacina, caracterizada pela ampla disseminação de informações falsas, com grande potencial para impactar a adesão da população à vacinação, especialmente após seu aumento significativo durante e pós-pandemia de covid-19,^25^ pode também ter contribuído para o cenário de menor cobertura vacinal entre as capitais da região Centro-Oeste.

Os resultados do presente estudo devem ser considerados à luz de suas limitações, como a não realização do censo demográfico em 2020, que obrigou o uso de dados antigos para a definição dos estratos socioeconômicos, o que pode ter alterado as comparações em algumas cidades onde a transformação urbana foi mais intensa. Os dados do nível familiar utilizados poderão auxiliar na identificação desses problemas até certo ponto, tendo em vista as limitações da classificação utilizada.^11^ A coleta de dados durante a pandemia de covid-19 também impactou as taxas de resposta. Ainda assim, destaca-se que o cálculo de pesos amostrais pós-estratificação contemplou diferenças nas respostas entre grupos populacionais e minimizou tais diferenças. Como pontos fortes, destacam-se o elevado tamanho amostral, além do rigor metodológico envolvido na coleta das informações vacinais.^11^ A tomada fotográfica das cadernetas, com posterior digitação dos dados por profissionais experientes no PNI, permitiu a excelente qualidade dessas informações.

No entanto, tais limitações não invalidam os resultados deste estudo, que apontam a grande heterogeneidade que existe na cobertura vacinal entre crianças de diferentes estratos sociais residentes nas capitais da região Centro-Oeste do Brasil. Além disso, observaram-se diferenças entre as maiores e menores coberturas por estrato, dentro das mesmas capitais. Também é importante enfatizar as baixas coberturas encontradas para a grande maioria das vacinas preconizadas até os 24 meses de vida. A investigação dos fatores intrínsecos às variáveis econômicas e sociais pode contribuir para uma intervenção assertiva e, consequentemente, melhorar os indicadores de imunização na região central do país. Portanto, há necessidade de abordagens direcionadas, considerando os estratos econômicos e vacinas com menor cobertura.
